# Functional trait analysis reveals the hidden stability of multitrophic communities

**DOI:** 10.1002/ecy.70001

**Published:** 2025-02-23

**Authors:** Mallarie E. Yeager, A. Randall Hughes

**Affiliations:** ^1^ Marine and Environmental Science, Marine Science Center Northeastern University Nahant Massachusetts USA; ^2^ Present address: Habitat Conservation Division, Alaska Region National Marine Fisheries Service, NOAA Juneau Alaska USA

**Keywords:** coastal fish communities, community stability, energy acquisition traits, functional traits, habitat filtering, locomotion traits, multitrophic communities, nutrient recycling traits

## Abstract

Although important for understanding how ecosystems will fare with increasing global change, the relationship between diversity and stability in multitrophic communities is still debated. Our best understanding comes from work within competitive guilds, where the relationship between stability and functional diversity is generally positive and also more direct and mechanistic than the relationship with species diversity. To expand our understanding, there is a need to examine empirically how functional trait identity relates to spatial and temporal stability within multitrophic communities relative to species identity. Here, we measured 13 functional traits of six coastal pond fish communities to examine temporal and spatial community stability through the lenses of functional trait diversity and species diversity. We found that solely considering species composition may underestimate stability. Additionally, we found spatial convergence and temporal divergence in species and trait variability, and we link this variation to processes of deterministic community assembly. Lastly, we found that correlations of species with key functional traits allow us to make inferences about how the trophic position of species relates to trait stability. Inferring community processes and making conservation decisions from species or trophic groups based on functional trait knowledge may be a viable strategy when resources are limited.

## INTRODUCTION

The rapid escalation of global change has made understanding the drivers of ecosystem stability a central issue for ensuring a sustainable future (Lade et al., 2019). Early ecological research hypothesized that biodiversity promotes stability, largely based on empirical observations that species‐poor communities such as those found in exploited agricultural systems tended to exhibit larger fluctuations in population size than species‐rich communities associated with natural systems such as old‐growth forests (Elton, 1958; MacArthur, 1955; Odum, 1959). However, theoretical work by May (1972) later contradicted this hypothesis by showing that in multitrophic communities constructed with randomized interaction strengths, complexity in the form of increased species diversity tended to destabilize ecosystems. This discrepancy between the relative stability of complex natural systems in the real world and their lack of stability in theoretical models led to a search for the existence of “stabilizing mechanisms” to help resolve this apparent complexity–stability paradox (McCann, 2000).

Although early studies of ecosystem stability focused on biodiversity measured as species richness, further work has shown that the sign and the magnitude of the effects of diversity on both functioning and stability are primarily driven by species distinctiveness in terms of functional roles or traits (Cadotte et al., 2011; Hooper et al., 2002; Mahaut et al., 2020). For instance, species richness often exhibits a saturating relationship with ecosystem function, as not every species added to the community contributes to a proportional increase in ecosystem function (Schwartz et al., 2000). However, when focusing explicitly on functional traits instead of species, the biodiversity–ecosystem function relationship becomes more linear, as each additional functional trait or group has less overlap with those already present (Cadotte et al., 2011). Therefore, focusing on species can underestimate the biodiversity–ecosystem function relationship, whereas focusing on functional traits can lead to a more direct and mechanistic foundation. This gain in understanding from a functional trait approach raises the question of how temporal change in functional traits relative to changes in species composition relates to the stability of a community.

The value of a functional trait–based approach for advancing community ecology was emphasized by McGill et al. (2006). Despite increases in understanding how functional diversity influences ecosystem processes, less is known about the stability of functional traits through space and time in multitrophic communities. Traditionally, species were categorized into functional groups based on similar roles or responses to the environment (Duffy et al., 2005; Hooper & Vitousek, 1997; Naeem et al., 2002; Tilman et al., 1997). To address concerns about how to assign species with continuous traits into categorical groups, how to integrate variation in species abundances, and how to resolve the inaccurate assumption that pairwise distance across traits is equivalent across all species within a functional group (Wright et al., 2006), metrics have been developed to allow for continuous traits (Mason et al., 2005; Petchey & Gaston, 2002) and analysis in multidimensional trait space (Laliberté & Legendre, 2010; Villéger et al., 2008). These multivariate functional trait indices provide a scheme for testing community stability as the turnover of functional traits through space and time in an ordination framework.

Visualizing patterns of community composition through time can reveal how individuals within a community assemble in mechanistic ways. Past work has hypothesized that communities assemble through niche‐based processes like habitat filtering (Keddy, 1992) and limiting similarity (MacArthur & Levins, 1967) or stochastic processes like neutral theory (Hubbell, 2011). Niche‐based theory of community assembly in n‐dimensional niche space predicts that habitat or environmental filtering should lead to species within a community exhibiting large overlap or higher redundancy in functional traits, whereas limiting similarity should result in more divergent patterns of functional traits. In plant communities, species tend to exhibit more similar types of traits, leading to habitat filtering as the main mechanism driving functional niche occupancy (Li et al., 2018). However, in multitrophic communities, there is evidence that limiting similarity explains species coexistence (Mason et al., 2008). When considering stability as temporal community turnover, comparing species compositional turnover to functional trait compositional turnover can inform whether community stability is accurately represented within species or whether high levels of redundancy due to habitat filtering create variation in species but not trait composition. Examining communities through a trait‐based lens in multivariate space thus allows us to test these ideas and compare spatial to temporal changes within species versus trait composition.

To expand our understanding of ecosystem stability, there is a need to understand empirically how functional trait identity relates to temporal stability within multitrophic communities relative to species identity. We examined compositional changes in a multitrophic fish community through time across an array of coastal ponds in Rhode Island at both the species and functional trait levels to test whether species compositional community stability is comparable to functional trait compositional community stability. We then identified which types of functional traits in fish consumer communities drive stability and examined whether we can make inferences about community stability using species identity by testing the correlation between functional traits and species composition. Lastly, by categorizing trophic positioning based on feeding mode, we tested whether specific trophic modes predict relationships between species and trait community stability.

## METHODS

### Study system

This study was conducted along the southern shore of Rhode Island across six coastal ponds: Green Hill (GH) Pond, Ninigret Pond (NP), Point Judith (PJ) Pond, Potter Pond (PP), Quonochontaug Pond (QP), and Winnapaug Pond (WP; Appendix S1: Figure S1). Fish communities in these ponds mostly consist of marine species connected to the ocean via a breachway that reassemble each year via larval dispersal from the ocean (Satchwill & Sisson, 1991a, 1991b; Sisson & Satchwill, 1991). The fish communities found throughout these ponds encompass a range of trophic levels (2–4.5) and feeding modes (Appendix S6: Table S1, Figure S1). Here, we examined 13 fish functional traits that can be categorized into three functional roles: (1) energy acquisition, (2) locomotion, and (3) nutrient recycling (Villéger et al., 2017). Fish communities are a good model system to ask these types of questions due to a strong foundation in the functional trait literature, offering a thorough understanding of the functional roles fish provide and functionally meaningful traits to measure (Albouy et al., 2011; Dumay et al., 2004; Mason et al., 2007; McLean et al., 2019; Mouillot et al., 2013; Stuart‐Smith et al., 2013; Villéger et al., 2010, 2017; Yeager et al., 2017).

### Fish community collections

From June to October 2018, we sampled six coastal pond fish communities monthly via a 46 m beach seine in conjunction with the Rhode Island Department of Environmental Management (RIDEM) fish and macroinvertebrate survey. This survey has been ongoing since 2010, with fish and macroinvertebrates identified to the lowest taxonomic level, enumerated and a subset measured to infer population size structure. Fish collections in 2018 for this study were collected via the same methods as the RIDEM survey. The fish in these communities span trophic level ranges from 2.1 to 4.5, consisting of detritivores, invertivores, and piscivores. We targeted 38 species that account for 99.4% of total abundance across the survey. For each species, we aimed to collect 20 individuals evenly distributed across their size range, informed by past survey data. Upon collection, the fish were either transferred into seawater containers for excretion incubations or euthanized immediately via a seawater‐clove oil (Eugenol extract, *Syzygium aromaticum*) mixture (IACUC protocol #: 18‐0622R). Once euthanized, the fish were held on ice before returning to the lab to conduct morphometric analysis. We collected a total of 708 fish across 27 species and 23 families. We analyzed a subset of 200 fish for nutrient recycling traits, resulting in an average of 18.63 ± 2.6 fish per species for energy acquisition and locomotion traits and an average of 7.48 ± 0.91 fish per family for nutrient recycling traits.

### Nutrient recycling traits

To quantify nutrient recycling traits, we conducted excretion incubations, targeting 27 fish families (*N* = 1–3 species per family), which account for 99.7% of total abundance across the survey. For each family, we targeted 10 individuals evenly distributed across their size range. Directly after fish were removed from the seine net, individuals were placed into separate 3 L sterile plastic bags of seawater directly taken from that site before seine collection and allowed to incubate for 30 min (Appendix S2: Figure S1f). During incubations, plastic bags were placed in a large cooler to ensure minimal stress. Directly after incubations, fish were transferred to a seawater–clove oil mixture for euthanasia. Two 60 mL 0.7 μm filtered water samples were taken from each plastic bag directly before and after each incubation trial, resulting in a pre‐ and post‐incubation water sample for both N and P concentrations. Water samples were placed on ice and frozen immediately once returning from the field and kept in a −20°C freezer until processing.

We analyzed all water samples for concentrations of ammonium (NH_4_^+^) and phosphate (PO_4_^3−^) using two spectraphotometric assays of phenolhypochlorite (Solórzano, 1969) and molybdenum blue (Murphy & Riley, 1962) methods modified by Whiles et al. (2009). To quantify the N and P contribution for each fish, we took the difference of N and P concentrations from the pre‐ and post‐incubation water samples (Appendix S2: Figure S1g). Lastly, we took the ratio of change of N to P for each fish to calculate the N:P functional trait.

### Morphological traits

To collect traits that inform energy acquisition and locomotion functional roles, we measured 15 morphometrics. For each fish, we took a series of five photos: (1) lateral full body, (2) lateral head, (3) lateral head with mouth protruded, (4) ventral full body, and (5) anterior with mouth open, all with a ruler in shot for length standardization (Appendix S2: Figure S1). Using the ImageJ analysis (Schneider et al., 2012), we measured 15 morphometrics that were used to calculate five energy acquisition traits: (oral gape surface, oral gape shape, oral gape position, protrusion, eye size) and five locomotion traits: (eye position, body transverse surface, body transverse shape, pectoral fin position, caudal peduncle throttling) (Appendix S2: Table S1a–e; Albouy et al., 2011). The 10 continuous functional trait measurements are commonly used in morphological studies on fishes, have been connected to diet or movement (Dumay et al., 2004; Mason et al., 2007; Sibbing & Nagelkerke, 2000; Villéger et al., 2010), and they are easily measured and broad enough to apply to any fish species.

### Generalized additive model fitting

We quantified the functional traits of fish from the past RIDEM fish and macroinvertebrate survey from 2010 to 2015 within the RI coastal ponds by fitting generalized additive models (GAMs) to the 13 functional traits (response variables) with species and length as predictor variables. GAMs model a response variable (e.g., functional trait) as the sum of nonlinear functions from different predictor variables (e.g., species and fork length; Hastie & Tibshirani, 1990). We utilized thin‐plate penalized regression splines, which add a penalty to the smoother function to avoid overfitting (Wood & Augustin, 2002). Penalty weights were optimized using the restricted maximum likelihood (REML) score which minimizes the root‐mean‐square error of the model fit to the data and balances model complexity with the goodness of fit and tends to undersmooth compared with other estimations like GCV. To run all models, we used the function “gam” of the mgcv R package (R Development Core Team, 2013). We used the GAM fit to each functional trait (Appendix S3 for model fits) to infer the functional traits of 27 fish species for which we had fork length data across 6 years and six ponds of survey data collected from RIDEM. Specifically, each year from 2010 to 2015, RIDEM sampled communities monthly from May to October via a 56 m beach seine net. Individuals were counted, measured, and identified to species. For the purpose of this study, we examined species and their abundances averaged across sampling stations (Appendix S1) to account for dependence within each pond and averaged across months to account for seasonal differences in species presence. This yielded functional trait information for a total of 81,337 fish.

### Relative stability analysis

**FIGURE 1:**
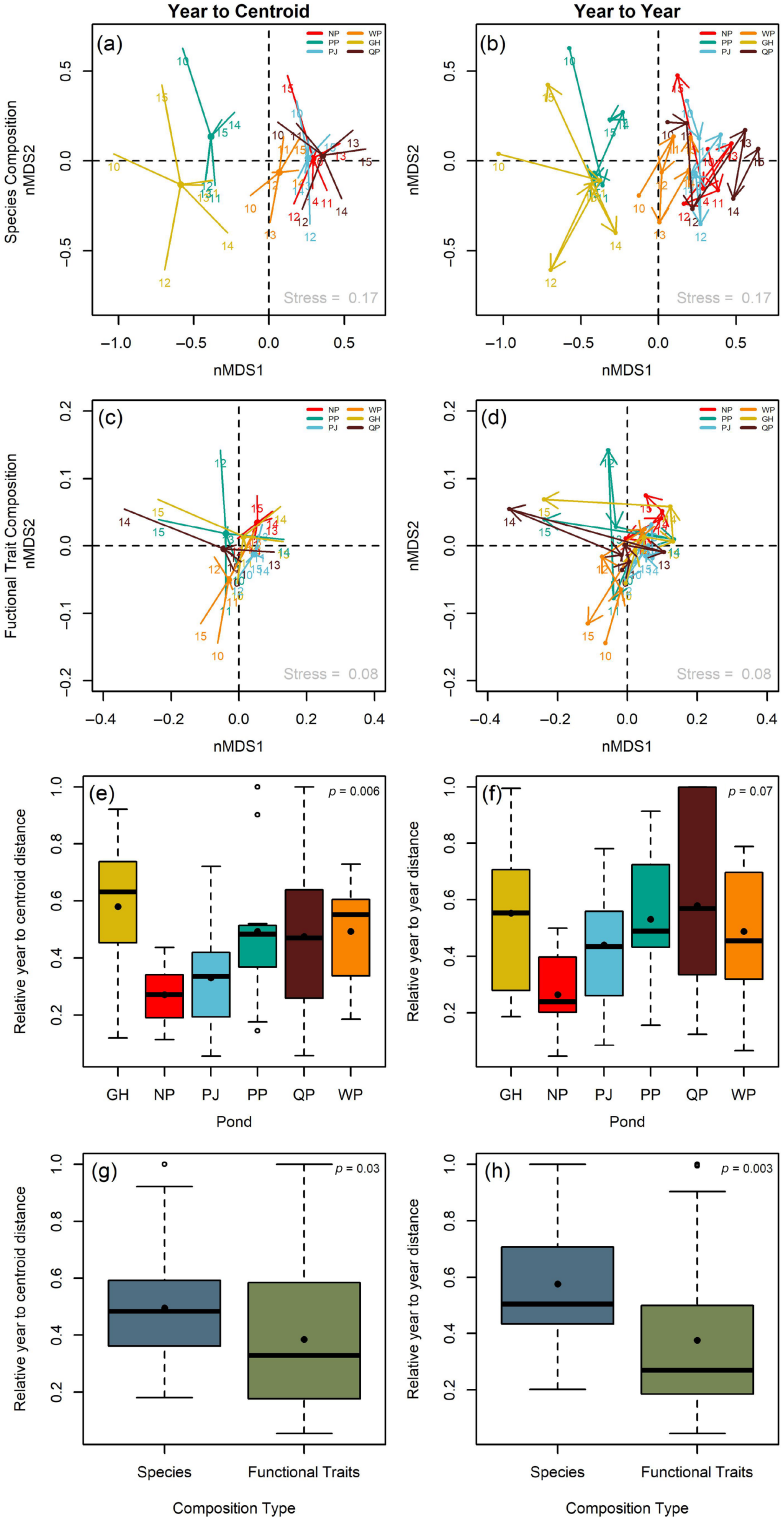
Relative community stability across space and time. Community ordinations across six ponds and 6 years by species (a, b) and functional trait composition (c, d). Community stability (smaller distances in ordination space = greater stability) was quantified for both composition types through the average year‐to‐centroid distance (a, c, e, g) and year‐to‐year distance (b, d, f, h) for each pond. Box plots (black point: Sample mean) showing relative stability across pond communities (e, f), and the type of composition (g, h) for both metrics. Coastal ponds: GH, Green Hill pond; NP, Ninigret pond; PJ, Point Judith pond; PP, Potter pond; QP, Quonochontaug pond; WP, Winnapaug pond. nMDS, nonmetric multidimensional scaling.

We examined the temporal stability across 6 years (2010–2015) of the six coastal pond fish communities and their functional traits in ordination space using nonmetric multidimensional scaling (nMDS). We constructed the functional trait matrix by calculating the community‐weighted values at each year by pond combination for each functional trait. We then used the “betadisper” function in the vegan R package on the dissimilarity matrix of both traits and species communities to calculate two metrics of community and trait stability: (1) average year‐to‐centroid distance and (2) average year‐to‐year distance. For both metrics, a lower value equates to greater stability via less community turnover. The average year‐to‐centroid distance is the average across all years of the Bray–Curtis dissimilarity between each pond community at each year and the pond community's average (or centroid; Figure 1a,c). This metric measures how the community diverges from its average composition throughout years. The second metric, average year‐to‐year distance, is the pond average of the Bray–Curtis dissimilarity between consecutive years (Figure 1b,d). This metric identifies small incremental changes. This metric is useful for evaluating whether a community is undergoing a directional shift due to an environmental change by comparing the consecutive year distances with the total change across all years. To compare the two stability metrics across species and trait community composition, we calculated the relative distance by dividing stability by the maximum distance found across all ponds and years for each composition type. We then ran a two‐way ANOVA for both year‐to‐centroid and year‐to‐year distance, testing whether relative stability differed across pond, trait versus species composition, or the interaction.

### Dissimilarity analysis

To test for statistical differences in community and trait composition across ponds and years, we used a permutational multivariate ANOVA (PERMANOVA). We then explored which traits and fish species were driving the dissimilarity across both year‐to‐centroid and year‐to‐year “distance” by conducting similarity percentage (SIMPER) analysis. For the year‐to‐centroid simper test, we first calculated the average species and trait values through time for each pond, then ran the simper analysis grouping by pond and selecting the comparisons of each year to the average. For the year‐to‐year simper test, we ran the simper analysis grouping by year and only selected the consecutive year comparisons. We used the functions “metaMDS,” “adonis,” and “simper” of the vegan R package to plot nMDS and calculate PERMANOVA, SIMPER, and the biplot correlation test, respectively (R Development Core Team, 2013). To examine how specific groups related to dissimilarity across ponds, we categorized species by feeding mode using FishBase.org (Froese & Pauly, 2023). The fish were assigned the following four feeding modes: (1) detritivore, (2) planktivore, (3) hunting meiofauna, and (4) hunting macrofauna (Appendix S6: Table S1).

### Biplot correlation analysis

Lastly, we examined the correlation of communities between functional traits and species and trophic composition. Using the function “envfit” of the vegan R package (R Development Core Team, 2013), we correlated the functional trait ordination with the species abundance matrix as well as a feeding mode abundance matrix. Plotting the species or trophic feeding mode whose abundances were significantly correlated with the ordination axes allowed us to identify species and trophic‐trait associations based on the overlap or the close proximity of species vectors with functional trait scores.

## RESULTS

### Relative stability analysis

For year‐to‐centroid relative stability, we found independent effects of pond and species versus trait composition, with no interaction between the two (Figure 1a,c,e,g; two‐way ANOVA: pond: *F*_5,60_ = 3.656, *p*‐value = 0.006, species vs. trait composition: *F*_1,60_ = 5.051, *p*‐value = 0.028, interaction: *F*_5,60_ = 0.318, *p*‐value = 0.901). Similarly, year‐to‐year distance showed independent effects of pond and species versus trait composition but no interaction (Figure 1b,d,f,h; two‐way ANOVA: pond: *F*_5,48_ = 2.224, *p*‐value = 0.067, species vs. trait composition: *F*_1,48_ = 10.107, *p*‐value = 0.003, interaction: *F*_5,48_ = 0.287, *p*‐value = 0.918). At the pond level, Ninigret was more stable than Green Hill Pond in year‐to‐centroid and more stable than Quonochontaug Pond in year‐to‐year distance, while all other ponds had no difference in relative stability. For both stability metrics, functional trait composition was more stable than species composition. As the species versus trait ordinations varied in the number of dimensions (27 species vs. 13 traits), we tested whether our findings of stability were an artifact of dimensional reduction. Specifically, we ran two simulation tests where we built mock communities of equivalent dimensions (simulation test 1: 13 species and 13 traits, and simulation test 2: eight species and eight traits) via random resampling of the community. We found similar results of higher functional trait composition stability than species composition stability in these simulations as in the realized Rhode Island fish community (Appendix S7), demonstrating that our findings are not a statistical artifact.

### Dissimilarity analysis

Functional traits varied across ponds (PERMANOVA: pond: *F*_5,24_ = 2.05, *p* = 0.05), but not across years (year: *F*_1,24_ = 2.31, *p* = 0.107), and there was no interaction between pond and year (interaction: *F*_5,24_ = 0.24, *p* = 0.99). Thus, functional trait composition varied significantly in space but was conserved through time. In contrast, species composition varied across both pond and year independently (PERMANOVA: pond: *F*_5,24_ = 1.84, *p* = 0.001; year: *F*_1,24_ = 3.09, *p* = 0.001), with no interaction (*F*_5,24_ = 0.81, *p* = 0.81), suggesting the relative species compositional differences across ponds remained consistent despite changes in species composition through time.

**FIGURE 2:**
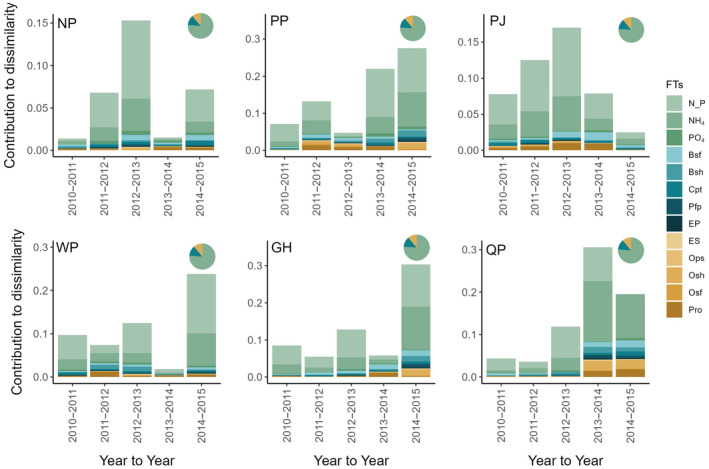
Spatiotemporal dissimilarity across functional trait communities. Stacked bar charts of the contribution of dissimilarity for each functional trait at each year‐to‐year time point across all six pond communities. The three groups of colors represent the three functional groups which the 13 functional traits inform: Energy acquisition (yellow), locomotion (blue), and nutrient recycling (green). Pie charts in the top left corner of each plot show the pond average dissimilarity contribution based on the three functional groups. Coastal ponds: GH, Green Hill pond; NP, Ninigret pond; PJ, Point Judith pond; PP, Potter pond; QP, Quonochontaug pond; WP, Winnapaug pond. Functional traits (FTs): Bsf, body surface; Bsh, body shape; Cpt, caudal peduncle throttling; EP, eye position; ES, eye size; N_P, N:P ratio; NH_4_, ammonium; Ops, oral position; Osf, oral surface; Osh, oral shape; Pfp, pectoral fin position; PO_4_, phosphate; Pro, protrusion.

Examining the SIMPER analysis of year‐to‐year distance for functional traits, we found that most of the dissimilarity across ponds was explained by nutrient recycling traits (77% of total dissimilarity across all ponds; Figure 2). That is, the variation in nutrient recycling traits within a community contributed the most to functional trait turnover. Locomotion traits made up the next highest contribution (~14% of total dissimilarity), followed by energy acquisition traits (~9% of total dissimilarity). We found similar results for year‐to‐centroid dissimilarity: 55% dissimilarity for nutrient recycling, 23% dissimilarity for locomotion, and 22% dissimilarity for energy acquisition traits (Appendix S4: Figure S1).

**FIGURE 3:**
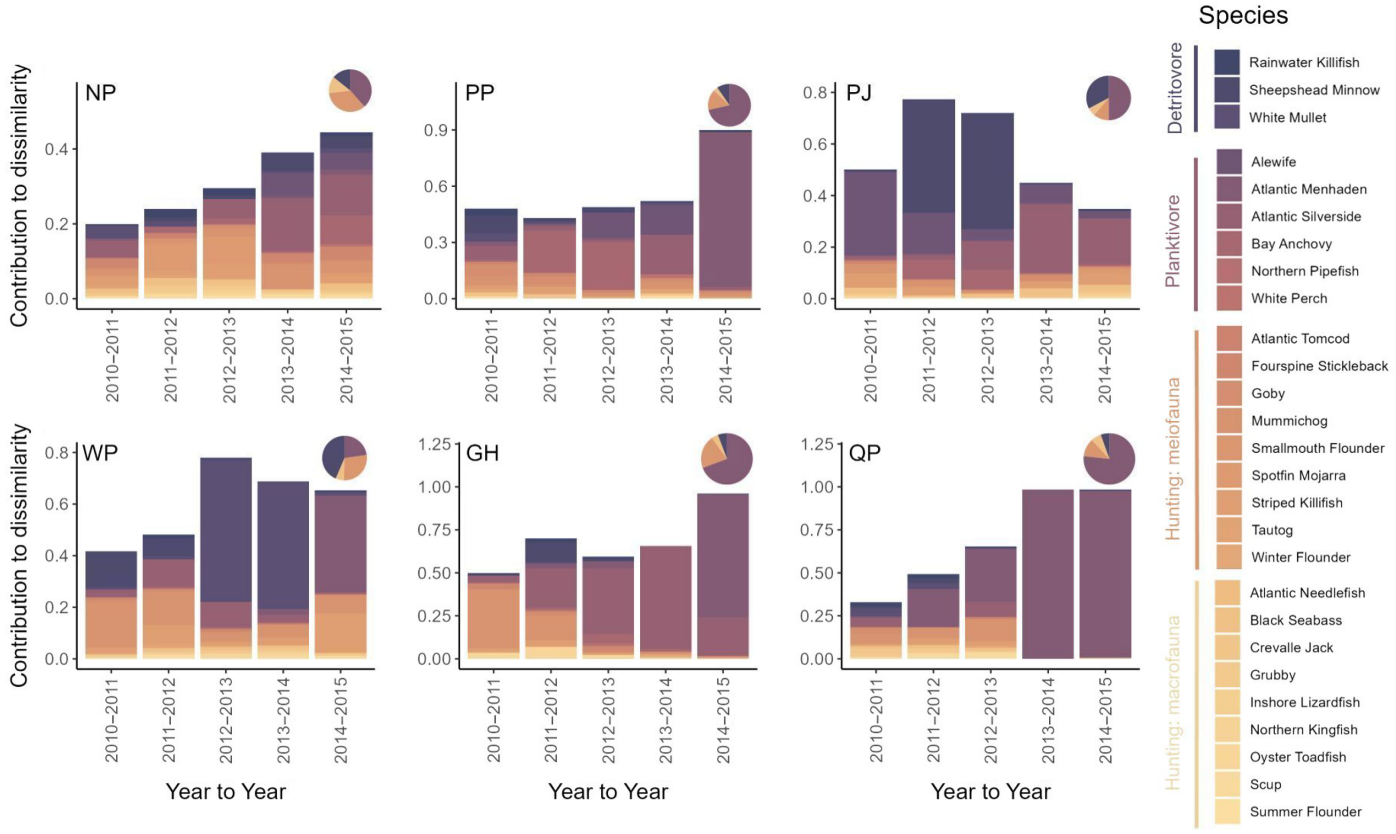
Spatiotemporal dissimilarity across species communities. Stacked bar charts of the contribution to dissimilarity for each fish species at each year‐to‐year time point across all six pond communities. The color legend on the left indicates the trophic group based on feeding mode. Pie charts in the top left corner of each plot show the pond average dissimilarity contribution based on the four groups: Dark purple—detritivore, dark pink—planktivore, orange—hunting predator: Meiofauna, and yellow—hunting predator: Macrofauna. Coastal ponds: GH, Green Hill pond; NP, Ninigret pond; PJ, Point Judith pond; PP, Potter pond; QP, Quonochontaug pond; WP, Winnapaug pond.

For species composition, the SIMPER analysis of year‐to‐year distance showed that a majority of the dissimilarity was due to species that can be categorized as planktivores (57% planktivore, 18% detritivore, 19% hunting meiofauna, 6% hunting macrofauna of the total dissimilarity across all ponds; Figure 3). The pond that showed the overall highest proportion of hunting meiofauna, Ninigret Pond, also had the highest stability values and overall lowest dissimilarity values. We found similar results when looking at year‐to‐centroid dissimilarity: 71% planktivore, 14% detritivore, 12% hunting meiofauna, and 3% hunting macrofauna (Appendix S4: Figure S2).

### Biplot correlation analysis

**FIGURE 4:**
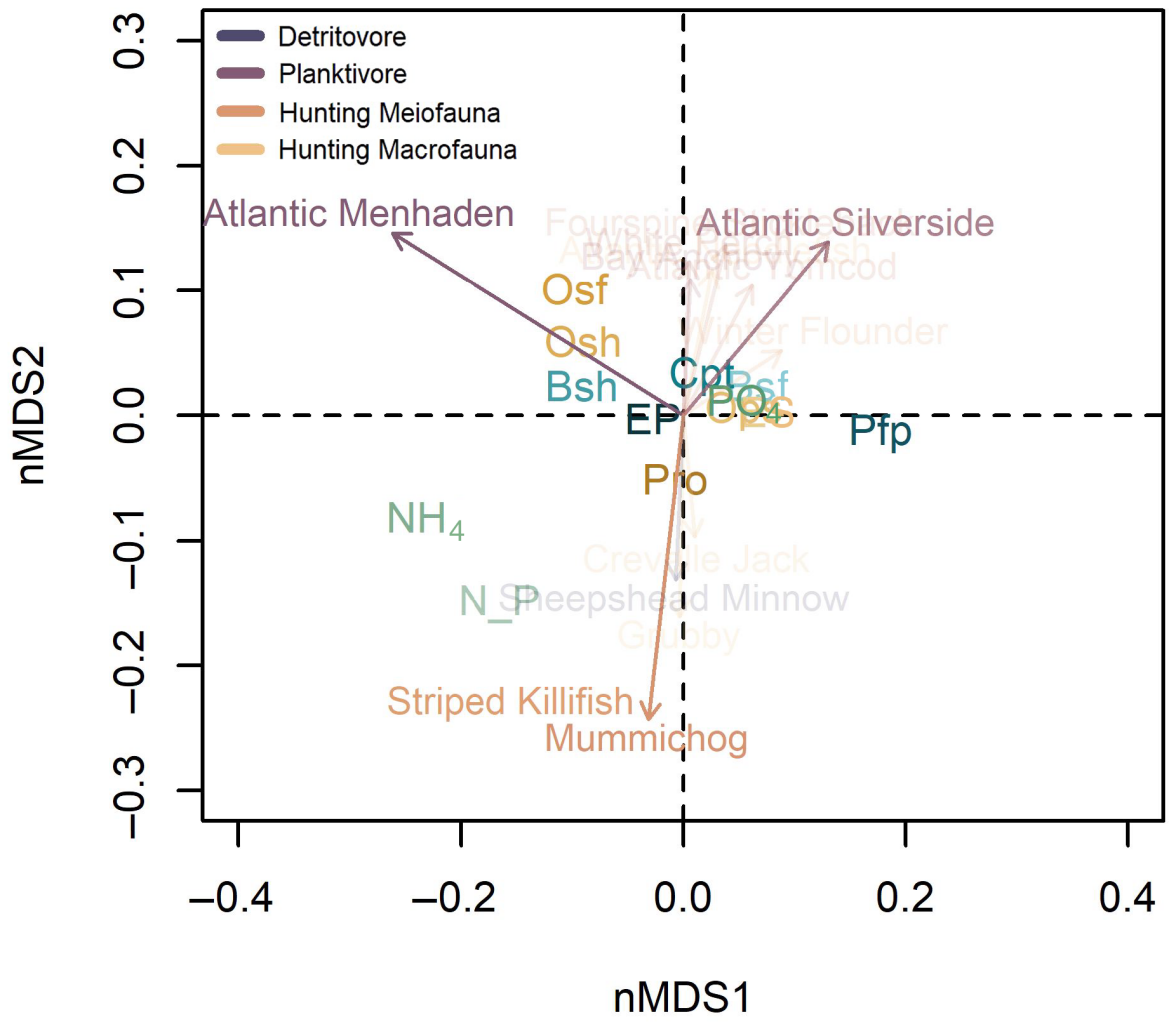
Biplot correlating the species community matrix with the functional trait community ordination. The functional trait scores are plotted on the ordination with overlaying correlating vectors of species. The black arrows indicate species vector correlations with the functional trait ordinations which had *p*‐value of ≥0.06 and the gray arrows are species, which had a *p*‐value of ≥0.1. All other species which had a *p*‐value <0.1 are vectors and are not shown. The colored arrows correspond to the trophic feeding mode vectors. Functional traits: Bsf, body surface; Bsh, body shape; Cpt, caudal peduncle throttling; EP, eye position; ES, eye size; N_P, N:P ratio; NH_4_, ammonium; Ops, oral position; Osf, oral surface; Osh, oral shape; Pfp, pectoral fin position; PO_4_, phosphate; Pro, protrusion. Vectors for Striped Killifish and Mummichog are overlapping. nMDS, nonmetric multidimensional scaling.

When we examined correlations between the functional trait ordination and the species abundance matrix, two planktivore species and two hunting meiofauna species were significantly correlated: (1) Atlantic menhaden (*Brevoortia tyrannus*), (2) Atlantic silverside (*Menidia menidia*), (3) mummichog (*Fundulus heteroclitus*), and (4) striped killifish (*Fundulus majalis*); Figure 4, Table 1. For the planktivores, Atlantic silverside had strong negative correlations with ammonium and N:P ratio, whereas Atlantic menhaden had slight negative correlations with phosphate and also positive correlations with energy acquisition traits (oral shape and surface) and the locomotion trait (body shape). The two species in the hunting meiofauna group, mummichog and striped killifish, were positively correlated with the energy acquisition trait protrusion (Pro).

**TABLE 1:** The statistical output from the correlations between the species community composition matrix and the functional trait community ordination.

Species	nMDS1	nMDS2	*R* ^2^	*p* value
Detritivore				
Rainwater killifish	−0.398	0.917	0.05	0.474
Sheepshead minnow	−0.054	−0.998	0.09	0.193
White mullet	0.630	0.777	0.01	0.770
Planktivore				
Alewife	0.730	0.684	0.03	0.46
Atlantic menhaden	−0.872	0.490	0.47	**0.0001**
Atlantic silverside	0.683	0.730	0.19	**0.07**
Bay Anchovy	0.055	0.998	0.06	0.310
Northern pipefish	−0.066	0.998	0.02	0.537
White perch	0.039	0.999	0.08	0.24
Hunting: Meiofauna				
Atlantic tomcod	0.508	0.861	0.08	0.243
Fourspine stickleback	0.264	0.965	0.10	0.152
Goby	−0.473	−0.881	0.01	0.859
Mummichog	−0.128	−0.992	0.32	**0.008**
Smallmouth flounder	0.481	0.877	0.01	0.75
Spotfin mojarra	0.350	0.937	0.01	0.91
Striped killifish	−0.125	−0.992	0.31	**0.009**
Tautog	0.486	−0.873	0.01	0.90
Winter flounder	0.861	0.509	0.05	0.35
Hunting: Macrofauna				
Atlantic needlefish	0.214	0.977	0.08	0.27
Black seabass	0.106	0.994	0.00	0.95
Crevalle jack	0.103	−0.995	0.05	0.40
Grubby	−0.023	−1.0	0.14	0.10
Inshore lizardfish	0.114	−0.993	0.03	0.63
Northern kingfish	0.976	0.220	0.01	0.82
Oyster toadfish	−0.327	0.945	0.02	0.718
Scup	0.159	−0.987	0.001	0.974
Summer flounder	0.169	0.986	0.03	0.647

*Note*: nMDS1 and nMDS2 show the vector directions, and bolded *p* values indicate significant correlations.

Abbreviation: nMDS, nonmetric multidimensional scaling.

When we looked at the feeding mode abundance matrix, the vectors of planktivore and hunting meiofauna were significantly correlated with the functional trait ordination (Appendix S6: Table S2, Figure S2). Both vectors correlated with energy acquisition traits. Specifically, the hunting meiofauna vector had a positive correlation with protrusion (Pro) and the planktivore vector had a positive correlation with oral shape (Osh) and oral surface (Osf), as well as body shape (Bsh; a locomotion trait).

## DISCUSSION

Our analysis of multitrophic fish communities through time and space corroborates calls to examine communities through a functional trait lens (McGill et al., 2006). We found temporal stability to be significantly higher when considering the community through a functional trait versus species composition perspective, even when dimensional reduction was accounted for (Appendix S7: Figure S1). These results demonstrate that the use of species composition may lead to underestimating underlying stability in the functional trait composition of communities. Furthermore, the convergence in species and trait variability through space, and their divergence in time, suggests that these pond communities are assembling in a deterministic way that can be explained through ecosystem and environmental filtering at varying scales (Tonn et al., 1990). Thus, the disconnect in our understanding of community stability derived from functional traits versus species composition may vary depending on the temporal and spatial scale examined. However, correlations of trophic groups within a community with key functional traits (e.g., planktivores with nutrient recycling traits) may allow us to make inferences about how groups of species relate to functional trait stability. Inferring community processes and making conservation decisions from species groups based on functional trait knowledge (i.e., functional groups) may be a viable strategy when resources are limited and exhaustively sampling continuous functional traits of entire communities is not possible.

### Functional redundancy

We found that measuring community stability using species identity underestimates the degree of stability in the functional composition of communities. This divergence in stability between species‐based metrics and trait‐based metrics is likely due to the level of functional redundancy, or the presence of multiple species within the community that exhibit similar functional traits (Lawton & Brown, 1994; Walker, 1992). Regardless of fluctuations in raw abundances, compensatory dynamics between functionally redundant species should buffer functional losses from any given species loss, leading to greater stability at the trait level (Biggs et al., 2020; Naeem, 1998; Petchey et al., 2007). For example, in the communities that were found to have the highest relative stability (NP and PJ), we found that although species abundances experienced inter‐annual variability, the total summed abundances across functional trophic feeding mode groups remained relatively consistent through time (Appendix S6: Figure S3). The presence of high functional redundancy can lead to an underestimation of the benefits of biodiversity for stability when measuring diversity at the species level, potentially resulting in misinformed management decisions to preserve biodiversity.

### Spatial versus temporal turnover

Inconsistencies between species and traits across space and time may be a result of the deterministic nature of how these communities assemble (Várbíró et al., 2020). Both functional traits and species were sensitive to changes across space in our study (Figures 2 and 3), suggesting that within‐pond environmental factors may shape these communities (Tonn et al., 1990). Although in close geographic proximity, the six ponds vary in their ecosystem characteristics, which may act as a filter on assembling species and traits, ultimately contributing to the differences across ponds (Mouillot et al., 2007; Tonn et al., 1990; Zobel, 1997). For example, pond size predicts community turnover at the species level, with less turnover and more species in larger ponds than in smaller ponds (Yeager et al., 2020). Many of these coastal ponds also have varying levels of biogenic habitats, like eelgrass (*Zostera marina*), salt marsh grass (*Spartina alterniflora*), and both natural and restored oyster reefs (*Crassostrea virginica*). This habitat identity and diversity can influence species and functional trait composition through species–specific habitat associations resulting from foraging (Boyd et al., 2015; Davoren et al., 2003; Yeager & Hovel, 2017), predator avoidance (Creel et al., 2005; Jordan et al., 1997; Wahle & Steneck, 1992), competition/territoriality (Rosenzweig, 1981), or reproduction (Wellenreuther & Clements, 2008) behaviors. Because these differences in key pond characteristics such as size and biogenic habitat remain consistent through time, they may cause or exacerbate the divergent spatial patterns in both functional trait and species composition.

In contrast to variation across space, functional trait turnover was not significant over time, despite temporal differences in species composition and the annual reassembly of a majority of the fish species in this system (Satchwill & Sisson, 1991a, 1991b; Sisson & Satchwill, 1991). These differences across space and time and between traits and species may be explained by the relationship of functional traits to species (Cadotte et al., 2011). If we assume that species identity is a product of the full complement or population of functional traits, then the composition of species will generally be expected to be more labile in space and time than the composition of a subset or sample of functional traits (Fonseca & Ganade, 2001). This is due to both dimensional reduction and asymmetrical mapping between species and functional traits (Appendix S7). Dimensional reduction is the consequence of only sampling a subset of the traits a species exhibits when using a functional trait approach (e.g., 3–5 traits for plant species but potentially more for consumer systems; Eklöf et al., 2013; Laughlin, 2014), thus likely only capturing a fraction of the variation in the total population of traits that characterize different species. This dimensional reduction then contributes to an asymmetrical one‐to‐many mapping from species to traits, whereby multiple species are likely to be associated with the same values for the subset of traits considered (Appendix S7: Figure S2b). This highlights the importance of considering which and how many traits to consider, as well as how broad a trait value spans across taxa (Lefcheck et al., 2015).

The combination of dimensional reduction and trait choice on the asymmetrical one‐to‐many mapping will tend to reduce the amount of turnover in composition observed when analyzing subsets of functional traits versus species. By building mock communities of equal numbers of species and traits via simulations, we controlled for dimensional reduction and still found functional trait community stability to be higher than species community stability (Appendix S7). Thus, we argue that the strong local filtering associated with persistent differences in key ecosystem characteristics (e.g., size and habitat) generates sufficiently high levels of spatial turnover so as to override the redundancy in functional traits produced via dimensional reduction and asymmetrical mapping, leading to significant variation in both species and functional trait composition. Conversely, because of their close geographical proximity and the relatively limited timespan of the study, these pond communities may have all experienced similar climatic forcing (Appendix S5), allowing the regional species pool to remain relatively consistent through time. If temporal differences are weak due to relatively consistent environmental conditions, then the redundancy provided by dimensional reduction and asymmetrical mapping would allow for trait composition, but not species composition, to remain consistent in time. Thus, variation in spatial and temporal filters on community assembly could mediate the relationship between species and functional trait composition turnover.

### Species groups can play a role in predicting community stability

Functional groups may provide a means of reconciling differences between inferences of community stability as measured by species versus continuous functional traits. Most species in the trophic group of planktivores are schooling, forage fish species. The strong correlation of forage fish with nutrient recycling traits like phosphate and the nitrogen: phosphorus ratio, as well as their importance to dissimilarity in species composition, supports the contribution of this trophic group to nutrient recycling trait variation. Forage fish act as an important subsidy in marine and freshwater systems, as they make up an extensive mobile biomass of bidirectional flows of energy, material, and organisms (Fox et al., 2018; Polis et al., 1997). For instance, alewife (*Alosa pseudoharengus*) was found to supply on average 1059 g of nitrogen and 120 g of phosphorus from marine systems into freshwater Bride Brook, Connecticut, each year, which enriched δ^15^N found in all stream trophic levels, suggesting the allochthonous input of the alewife subsidy (Walters et al., 2009). Future studies interested in the role of nutrient recycling traits in coastal fish communities can use prior knowledge of forage fish as subsidies to inform their functional impact on the ecosystem.

Continuous metrics of functional traits allow for more fine‐scale measurements of functional diversity at the individual level and, in this study, revealed greater stability at the community level than species identity. The divergence of species and traits relative to stability supports the notion of moving toward a trait‐based world in community ecology. However, when data limitations exist or resources are low, integrating functional trait knowledge of well‐studied communities, like the functional traits databases of fish (Lecocq et al., 2019) or plant communities (Kattge et al., 2011), to make inferences about functional groups of species, may allow researchers to make better predictions about community stability and thus bridge the gap between traditional and trait‐based approaches. Understanding the relationship between biodiversity and stability, through both trait and species lens, will be critical to predicting how communities will respond to global change.

## AUTHOR CONTRIBUTIONS

Mallarie E. Yeager and A. Randall Hughes designed the study. Mallarie E. Yeager analyzed the data and wrote the first draft of the manuscript. A. Randall Hughes contributed to all subsequent drafts.

## CONFLICT OF INTEREST STATEMENT

The authors declare no conflicts of interest.

## Supporting information


Appendix S1.

Appendix S2.

Appendix S3.

Appendix S4.

Appendix S5.

Appendix S6.

Appendix S7.

## Data Availability

Fish community data (Yeager and Hughes, 2020) are available in the Biological and Chemical Oceanography Data Management Office (BCO‐DMO) repository at https://doi.org/10.1575/1912/bco-dmo.805252.1. Functional trait data (Hughes and Yeager, 2022) are available in the BCO‐DMO repository at https://doi.org/10.26008/1912/bco-dmo.870857.2. Additional data, scripts and workflow (Yeager, 2025) are available in Zenodo at https://doi.org/10.5281/zenodo.14597555.
