# Supplementary material for: Functional trait analysis reveals the hidden stability of multitrophic communities
Source: Ecology. 2025 Feb 23;106(2):e70001. doi: 10.1002/ecy.70001 (PMC11848122; doi:10.1002/ecy.70001)

Yeager, M.E., Hughes, A.R. Functional trait analysis reveals the hidden stability of multitrophic communities. Ecology

Appendix S1. Map of Rhode Island Coastal Ponds

Figure S1. Map of the six coastal ponds on the south shore of Rhode Island and the respective sites within each pond where community fish data was sampled. Coastal ponds: NP = Ninigret pond, PP = Potter pond, PJ = Point Judith pond, WP = Winnapaug pond, GH = Green Hill pond, QP = Quonochontaug pond.

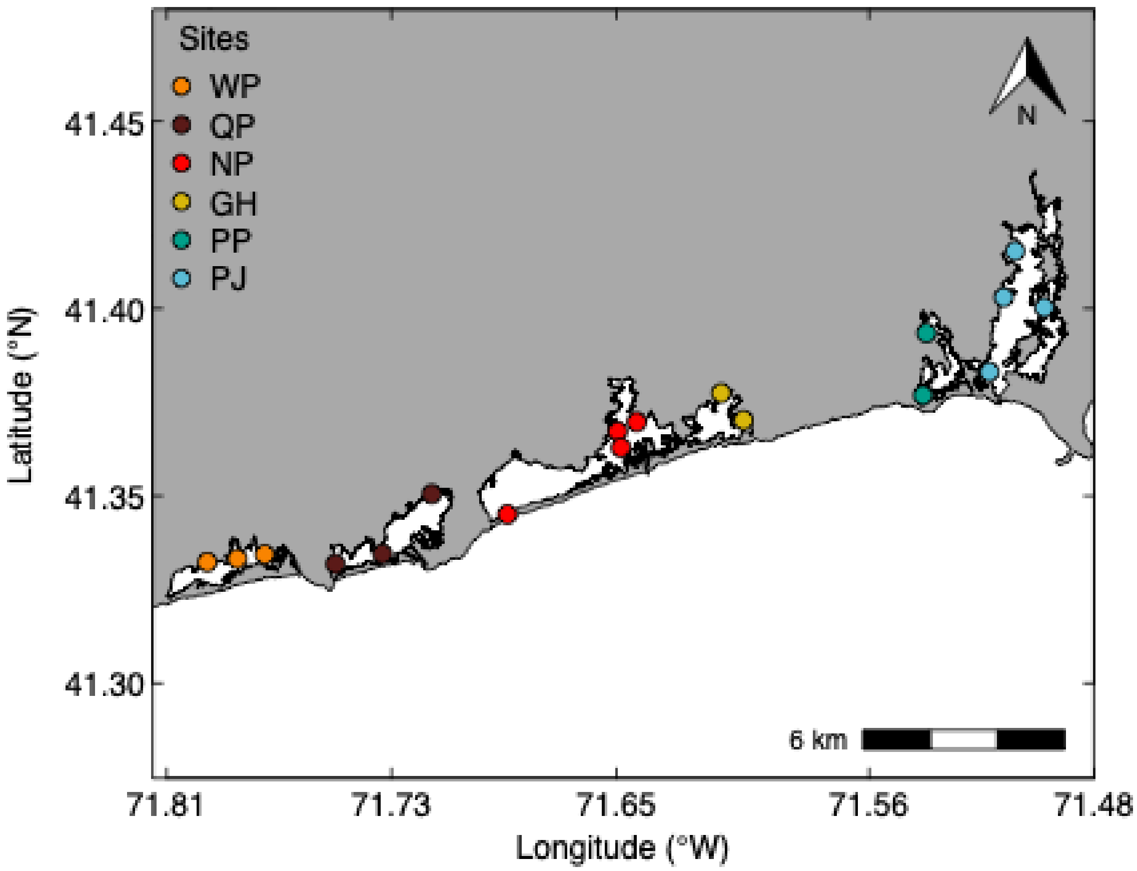

Supplement: Supplementary file 1 — Appendix S1. [file ECY-106-e70001-s006.pdf]
