# Supplementary material for: Functional trait analysis reveals the hidden stability of multitrophic communities
Source: Ecology. 2025 Feb 23;106(2):e70001. doi: 10.1002/ecy.70001 (PMC11848122; doi:10.1002/ecy.70001)
Supplement: Supplementary file 2 — Appendix S2. [file ECY-106-e70001-s003.pdf]

Yeager, M.E., Hughes, A.R. Functional trait analysis reveals the hidden stability of multitrophic communities. Ecology

Appendix S2. Quantifying morphology and nutrients for functional trait calculations

Figure S1. Example photos of the 15 morphometrics measured in this study (a-e). The abbreviations correspond to the equations in Table 1. Photos of the excretion assay (f) the in-field excretion incubations and (g) ammonium molybdenum blue spectra-photometric assays pre- and post-incubation on the same sample. The colored boxes relate to the type of functional role each measurement contributes to (yellow: energy acquisition, blue: locomotion, green: nutrient recycling) and the grey boxes (SL – standard length, FL – fork length) used for GAM models. Photo credits: Mallarie E. Yeager.

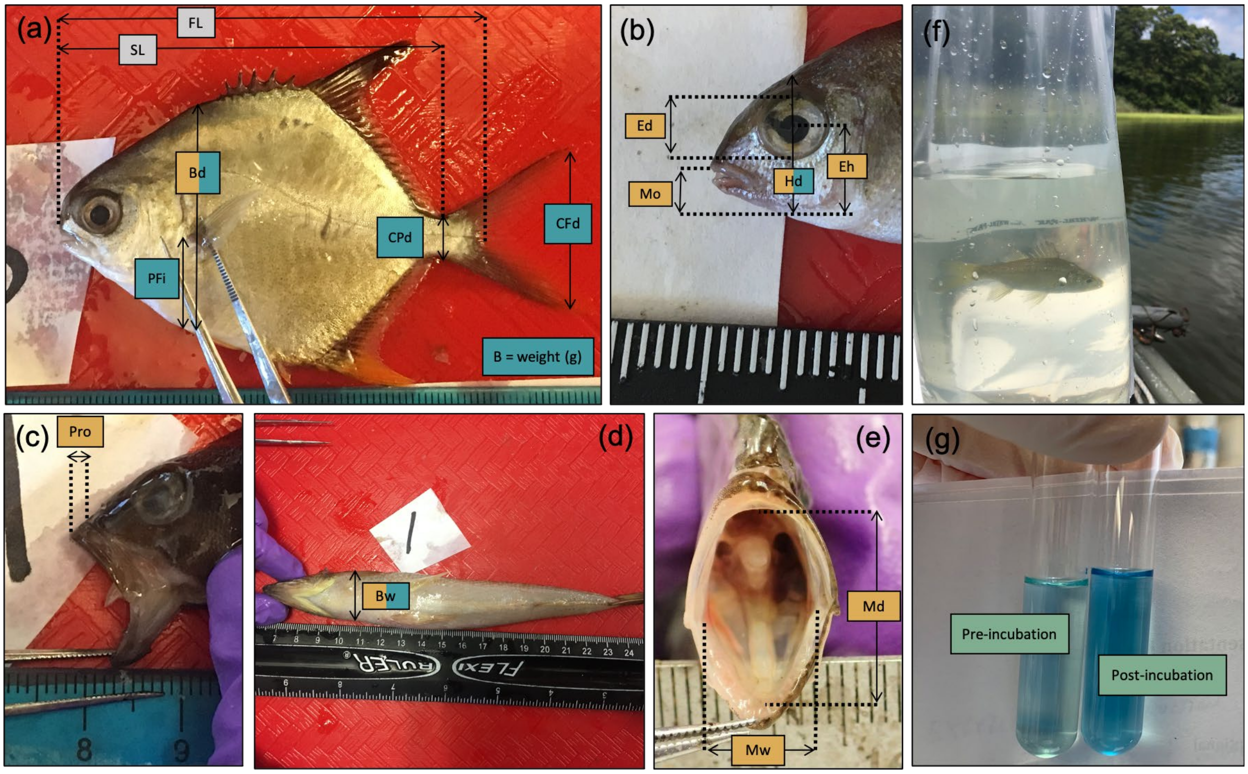

Table S1. Functional trait equations based on the 13 morphological traits measured on each fish.

Abv.	Functional Trait	Equation
	Energy acquisition traits	
Osf	Oral gape surface	$\frac{Mw \times Md}{Bw \times Bd}$
Osh	Oral gape shape	$\frac{Md}{Mw}$
Ops	Oral gape position	$\frac{Mo}{Hd}$
Pro	Protrusion	Pro
Es	Eye size	$\frac{Ed}{Hd}$
	Locomotion traits	
Ep	Eye position	$\frac{Eh}{Hd}$
Bsh	Body transversal shape	$\frac{Bd}{Bw}$
Bsf	Body surface	$\frac{\ln\left(\left(\frac{\pi}{4} \times Bw \times Bw\right) + 1\right)}{\ln(B + 1)}$
Pfp	Pectoral fin position	$\frac{PFi}{PFb}$
Cpt	Caudal peduncle throttling	$\frac{CFb}{CPd}$
