# Supplementary material for: Functional trait analysis reveals the hidden stability of multitrophic communities
Source: Ecology. 2025 Feb 23;106(2):e70001. doi: 10.1002/ecy.70001 (PMC11848122; doi:10.1002/ecy.70001)

Yeager, M.E., Hughes, A.R. Functional trait analysis reveals the hidden stability of multitrophic communities. Ecology

Appendix S3. Generalized Additive Model summary statistics

Method

Generalized additive models (GAMs) were fit on the collected fish functional traits. Below, are the GAM fits for each trait model (Table S1) and plots visualizing the fits (Figure S1). When using GAMs to predict functional trait onto past community survey data, we constrained the data to the size range that our functional traits data was measured (e.g., for Black Seabass we collected fish that ranged from 38-94mm so the community data for both traits and species were augmented to only consider Black Seabass in that range). The functional trait PO4 had some species with measurements of 0, which led to poor fitting models. For this trait when a fish had a zero measurement, we instead calculated the mean PO4 and did not incorporate length. For fish which did not have any measurements of 0, we fit the GAM model the same way we did the other FTs.

Table S1. The generalized additive model (GAM) fits for all 13 functional traits. GCV = generalized cross validation score. * PO4 models only run on species which do not have values of zero (see methods).

Model	Family	R^2	Deviance explained
Osf ~ species + s(length, by = species)	gaussian	0.90	0.91
Osh ~ species + s(length, by = species)	gaussian	0.70	0.73
Ops ~ species + s(length, by = species)	gaussian	0.40	0.46
Pro ~ species + s(length, by = species)	tweedie	0.80	0.84
ES ~ species + s(length, by = species)	gaussian	0.69	0.72
EP ~ species + s(length, by = species)	gaussian	0.74	0.77
Bsh ~ species + s(length, by = species)	gaussian	0.89	0.91
Bsf ~ species + s(length, by = species)	gaussian	0.94	0.95
Pfp ~ species + s(length, by = species)	gaussian	0.86	0.87
Cpt ~ species + s(length, by = species)	gaussian	0.62	0.66
NH4 ~ species + s(length, by = species)	gaussian	0.44	0.60
PO4 ~ species + s(length, by = species)*	gaussian	0.24	0.45

Figure S1. GAM fit plots of biomass and the 13 functional traits by length of each fish (fork length). Points correspond to a measured data point of each fish while the lines represent the GAM fits. Each color is a different species (morphometrics) or family (nutrient recycling traits).

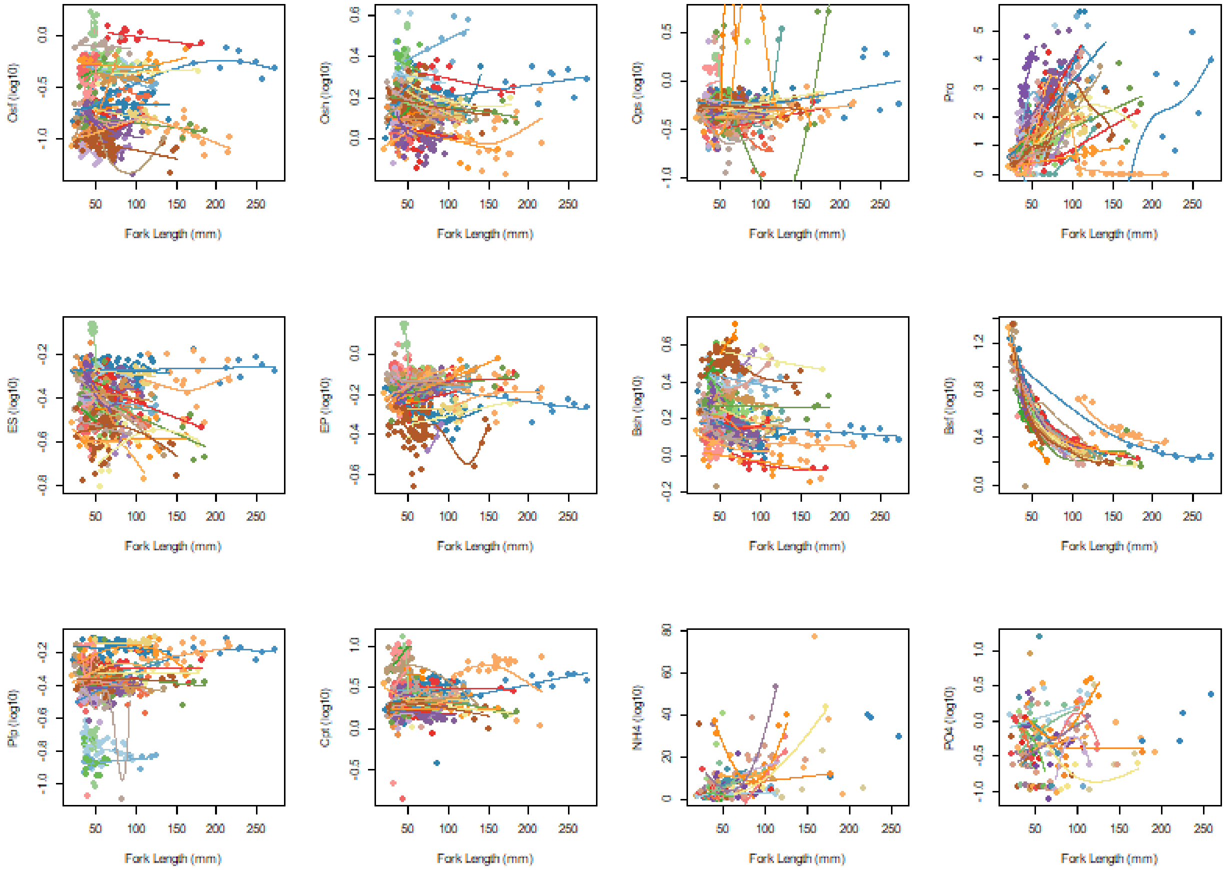

Supplement: Supplementary file 3 — Appendix S3. [file ECY-106-e70001-s001.pdf]
