# Supplementary material for: Functional trait analysis reveals the hidden stability of multitrophic communities
Source: Ecology. 2025 Feb 23;106(2):e70001. doi: 10.1002/ecy.70001 (PMC11848122; doi:10.1002/ecy.70001)

Yeager, M.E., Hughes, A.R. Functional trait analysis reveals the hidden stability of multitrophic communities. Ecology

Appendix S4. Year-to-centroid community dissimilarity

Figure S1. Spatiotemporal dissimilarity across functional trait communities. Stacked bar charts of the contribution of dissimilarity for each functional trait at each year-to-centroid time point across all six pond communities. The three groups of colors represent the three functional groups which the 13 functional traits inform: energy acquisition (yellow), locomotion (blue) and nutrient recycling (green). Pie charts in the top left corner of each plot show the pond average dissimilarity contribution based on the three functional groups. Coastal ponds: NP = Ninigret pond, PP = Potter pond, PJ = Point Judith pond, WP = Winnapaug pond, GH = Green Hill pond, QP = Quonochontaug pond. Functional traits: N_P = N: P ratio, NH4 = ammonium, PO4 = phosphate, Bsf = body surface, Bsh = body shape, Cpt = caudal peduncle throttling, Pfp = pectoral fin position, EP = eye position, ES = eye size, Ops = oral position, Osh = oral shape, Osf = oral surface, Pro = protrusion.

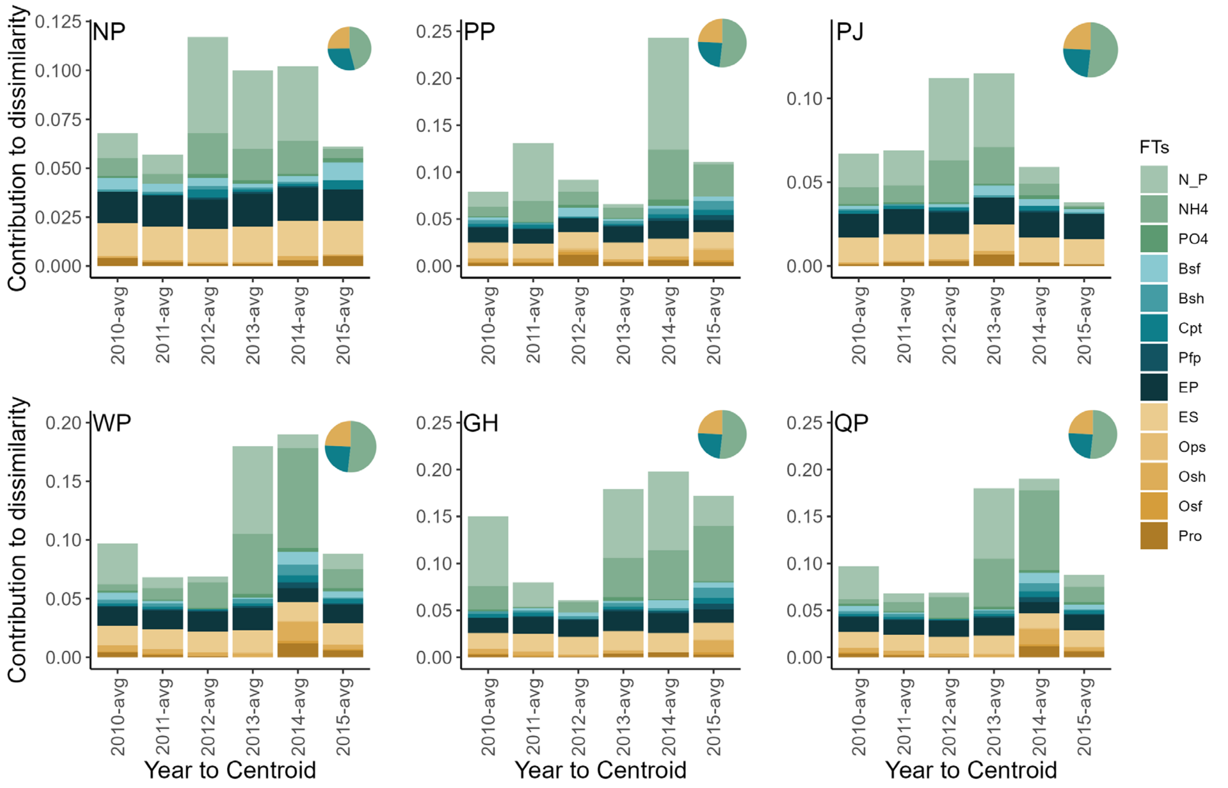

Figure S2. Spatiotemporal dissimilarity across species communities. Stacked bar charts of the contribution of dissimilarity for each fish species at each year-to-centroid time point across all six pond communities. The color legend on the left indicates the trophic group based on feeding mode. Pie charts in the top left corner of each plot show the pond average dissimilarity contribution based on the four groups: dark purple – detritivore, dark pink – planktivore, orange – hunting predator: meiofauna, and yellow – hunting predator: macrofauna. Coastal ponds: NP = Ninigret pond, PP = Potter pond, PJ = Point Judith pond, WP = Winnapaug pond, GH = Green Hill pond, QP = Quonochontaug pond.

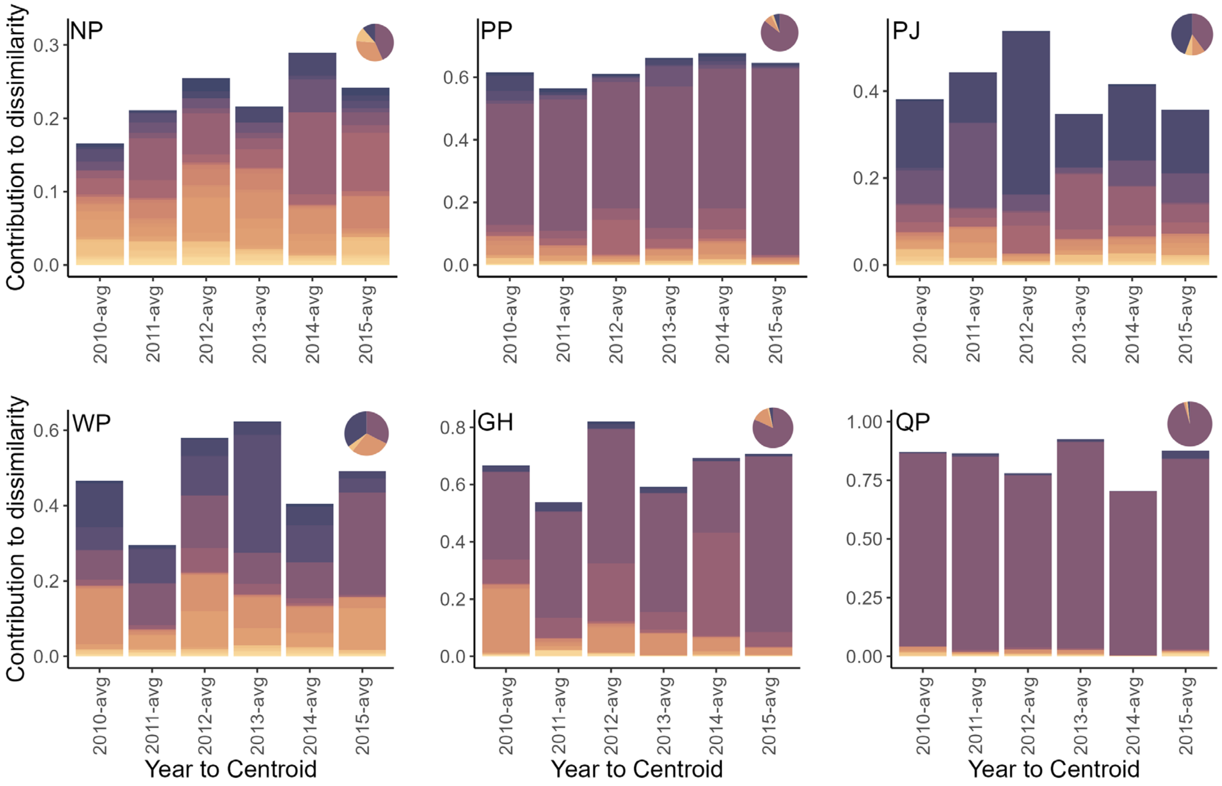

Supplement: Supplementary file 4 — Appendix S4. [file ECY-106-e70001-s004.pdf]
