# Supplementary material for: Functional trait analysis reveals the hidden stability of multitrophic communities
Source: Ecology. 2025 Feb 23;106(2):e70001. doi: 10.1002/ecy.70001 (PMC11848122; doi:10.1002/ecy.70001)
Supplement: Supplementary file 5 — Appendix S5. [file ECY-106-e70001-s007.pdf]

Yeager, M.E., Hughes, A.R. Functional trait analysis reveals the hidden stability of multitrophic communities. Ecology

Appendix S5. Climate factors in Rhode Island over the study timeline

The sea surface temperature metrics (monthly mean, min and max) in the coastal waters off Rhode Island shows one example of consistent environmental conditions over the study timeline. This lack of abrupt of unusual temporal change in environmental conditions might explain the limited variation in functional traits observed through time, as the regional species pool likely remained relatively consistent through time. This could underpin why interspecific functional redundancy yielded temporally invariant, but spatially distinct functional trait structure across ponds via distinct within-pond environmental characteristics.

Figure S1. Average monthly mean sea surface temperature (°C) for the coastal waters around the Rhode Island coastal ponds. SST data downloaded from NOAA Coral Reef Watch (Liu et al., 2009) which is a daily global 5km satellite product. We calculated mean monthly SST at -71.725 W, 41.375 N ± 0.05 degrees.

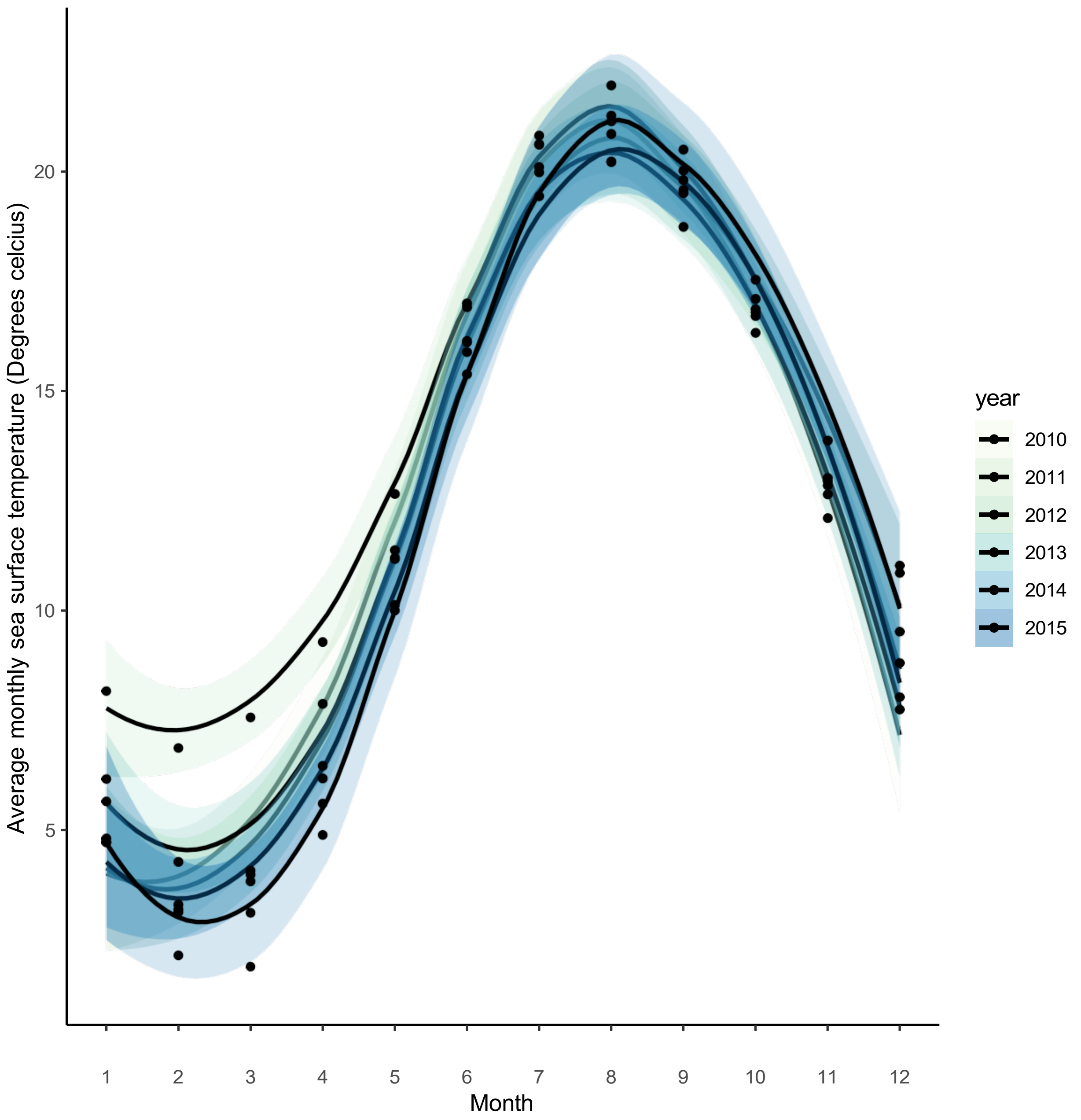

Figure S2. Average monthly minimum sea surface temperature (°C) for the coastal waters around the Rhode Island coastal ponds. SST data downloaded from NOAA Coral Reef Watch (Liu et al., 2009) which is a daily global 5km satellite product. We calculated minimum monthly SST at -71.725 W, 41.375 N ± 0.05 degrees.

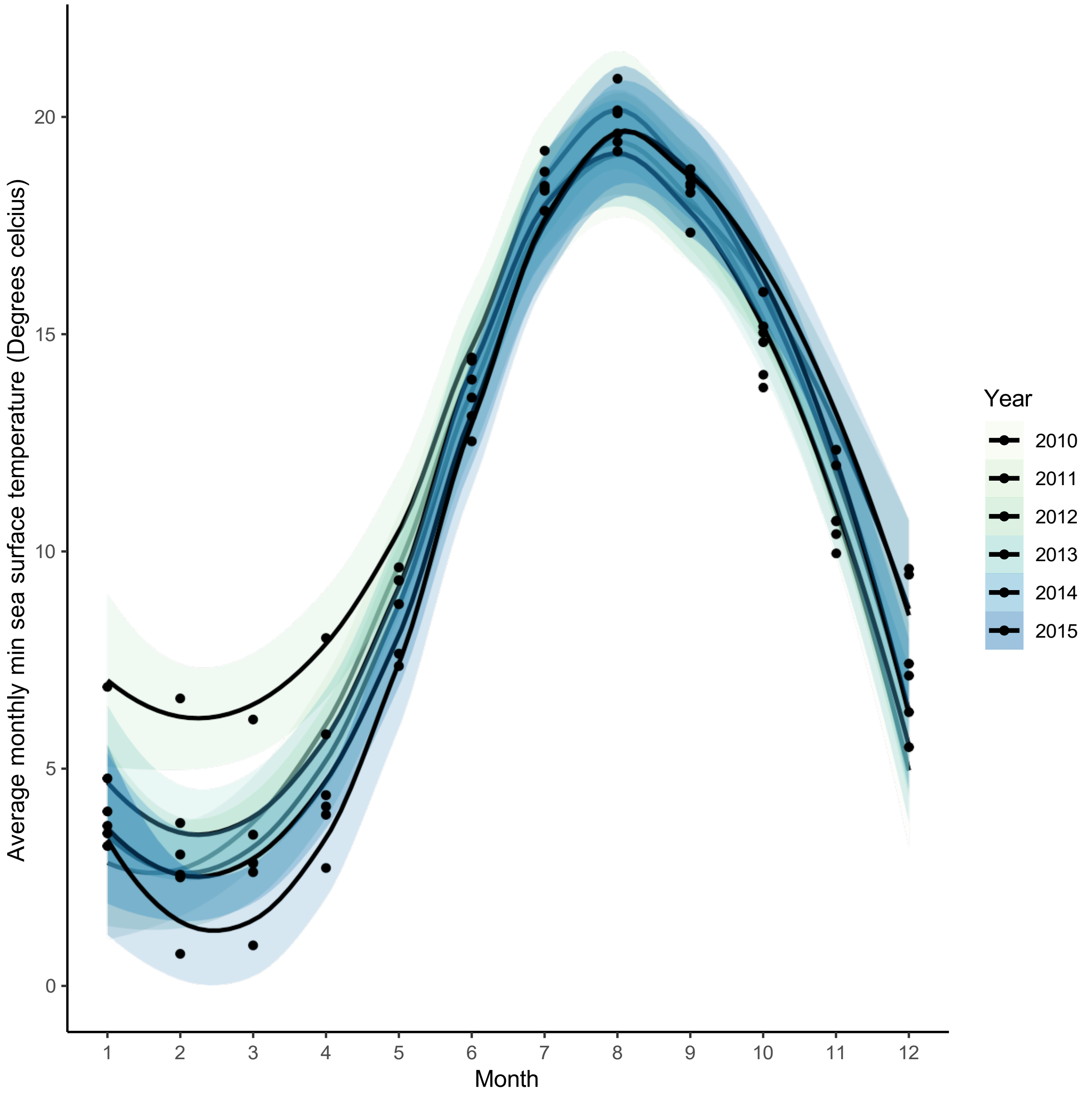

Figure S3. Average monthly maximum sea surface temperature (°C) for the coastal waters around the Rhode Island coastal ponds. SST data downloaded from NOAA Coral Reef Watch (Liu et al., 2009) which is a daily global 5km satellite product. We calculated maximum monthly SST at -71.725 W, 41.375 N ± 0.05 degrees.

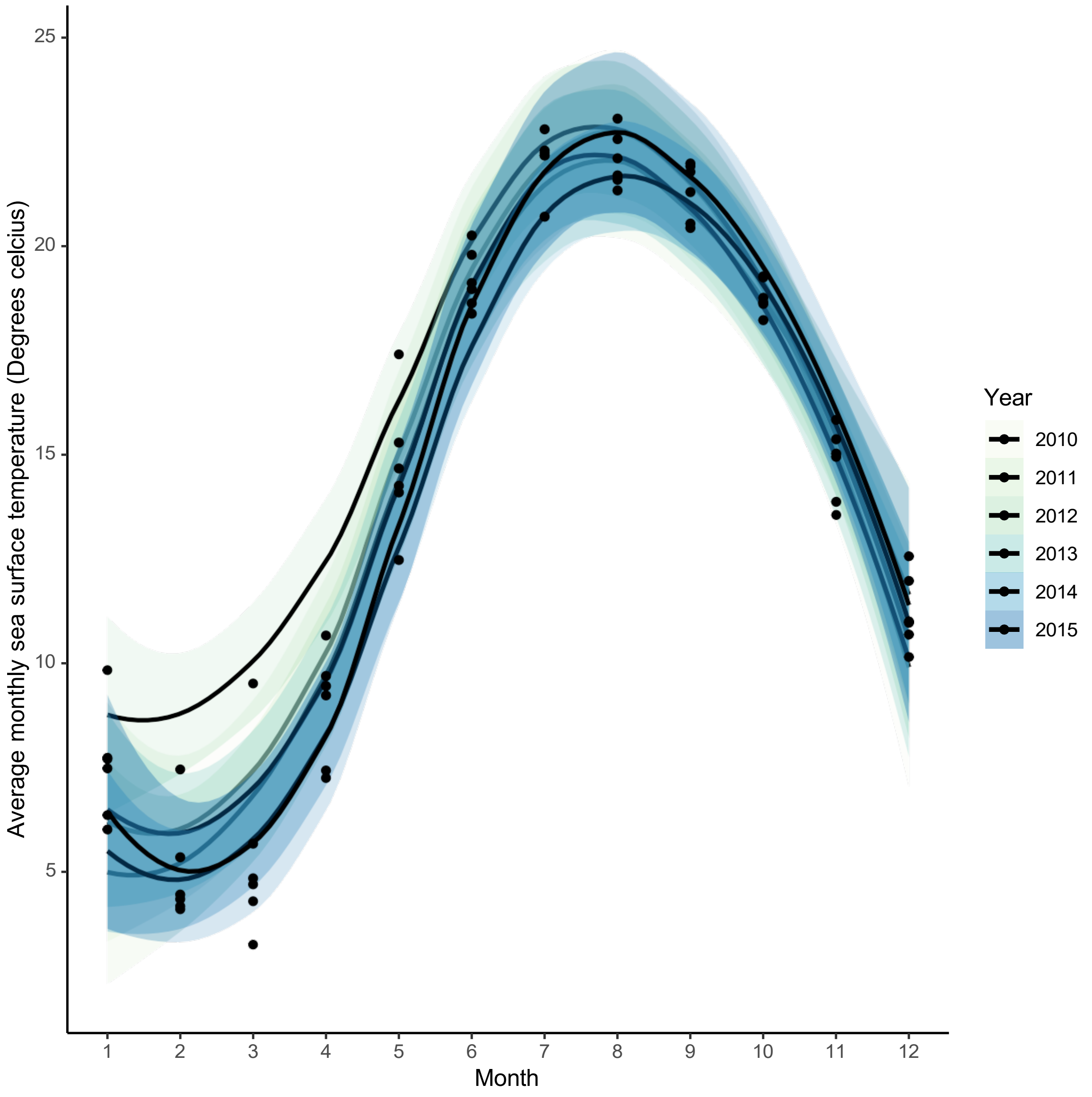

References

Liu, Gang; Heron, Scott F.; Eakin, C. Mark; De La Cour, Jacqueline L.; Geiger, Erick F.; Tirak, Kyle V.; Skirving, William J.; Strong, Alan E. (2018). NOAA Coral Reef Watch (CRW) Daily Global 5-km (0.05 degree) Satellite Coral Bleaching Heat Stress Monitoring Product Suite. [Mean monthly SST 2010:2015]. NOAA National Centers for Environmental Information. Dataset. https://doi.org/10.25921/6jgr-pt28. Accessed 05/19/2022.
