# Supplementary material for: Functional trait analysis reveals the hidden stability of multitrophic communities
Source: Ecology. 2025 Feb 23;106(2):e70001. doi: 10.1002/ecy.70001 (PMC11848122; doi:10.1002/ecy.70001)
Supplement: Supplementary file 6 — Appendix S6. [file ECY-106-e70001-s002.pdf]

Yeager, M.E., Hughes, A.R. Functional trait analysis reveals the hidden stability of multitrophic communities. Ecology

Appendix S6. Trophic Structure

Figure S1: Density plots of the trophic structure based on the composition of trophic levels across the 6 coastal ponds (colors) throughout the six years (panels).

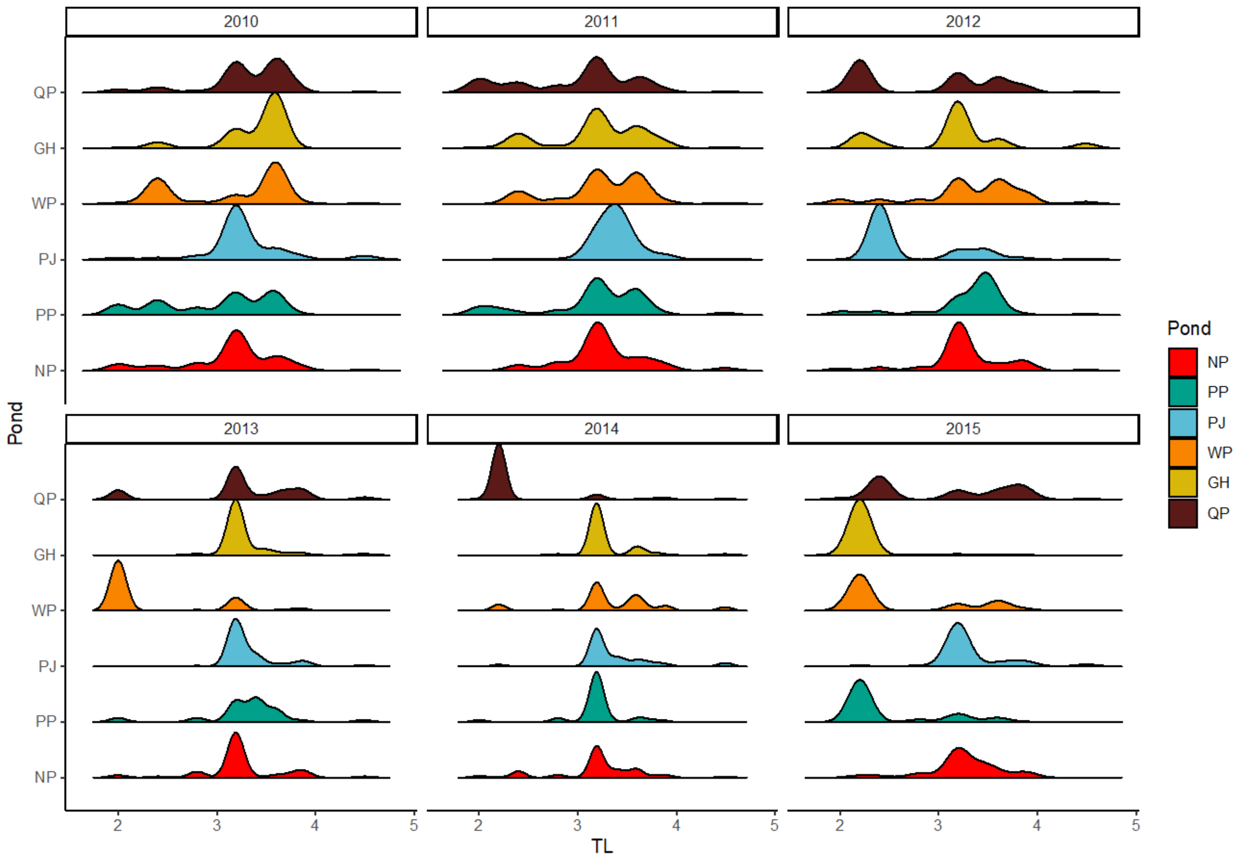

Table S1. Trophic information on fish species in this study. Constructed from fishbase.org (Froese and Pauly, 2023) and the references within.

**Common Name**	**Scientific Name**	**Trophic Level**	**Feeding Mode**	**Primary Prey Type**	**Secondary Prey Type**	**Reference**
White Mullet	*Mugil crema*	2.00	detritovore	Detritus	Algae	(Gómez-Canchong et al.,2004)
Atlantic Menhaden	*Brevoortia tyrannus*	2.20	planktivore	Krill	Amphipods	(Lewis and Peters 1994)
Sheepshead Minnow	*Cyprinodon variegatus*	2.40	detritovore	Detritus	Algae	(Bowman 2000)
Rainwater Killifish	*Lucania parva*	2.80	detritovore	Copepods	Polychaetes	(Harrington and Harrington 1961)
White Perch	*Morone americana*	3.10	planktivore	Cladocerans	Zooplankton	(Prout et al. 1990)
Northern Pipefish	*Syngnathus fuscus*	3.17	planktivore	Mysid shrimp	Amphipods	(Bowman 2000)
Atlantic Silverside	*Menidia menidia*	3.19	planktivore	Copepods	Mysid shrimp	(Adams 1976)
Fourspine Stickleback	*Apeltes quadracus*	3.20	Hunting: meiofauna	Diatoms	Marine worms	(Murdy et al. 1997)
Goby	*Gobiosoma bosci*	3.20	Hunting: meiofauna	Polychaetes	Amphipods	(Bowman 2000)
Spotfin Mojarra	*Eucinostomus argenteus*	3.24	Hunting: meiofauna	Copepods	Amphipods	(Austin and Austin 1971)
Atlantic Tomcod	*Microgadus tomcod*	3.30	Hunting: meiofauna	Copepods	Amphipods	(Grabe 1978)
Tautog	*Tautoga ontis*	3.32	Hunting: meiofauna	Copepods	Shrimp	(Leim 1966)
Alewife	*Alosa pseudoharengus*	3.40	planktivore	Amphipods	Shrimp	(Bowman 2000)
Smallmouth Flounder	*Etropus microstomus*	3.40	Hunting: meiofauna	Polychaetes	Molluscs	(José and Olinto 2003)
Striped Killifish	*Fundulus majalis*	3.40	Hunting: meiofauna	Polychaetes	Molluscs	(Bowman 2000)
Bay Anchovy	*Anchoa michilli*	3.46	planktivore	Copepods	Barnacle larvae	(Allen et al. 1995)a
Crevalle Jack	*Caranx hippos*	3.56	planktivore	Fish	Shrimp	(Gómez-Canchong et al.,2004)
Northern Kingfish	*Menticirrhus saxatilis*	3.56	planktivore	Decapods	Fish	(Bowman 2000)
Mummichog	*Fundulus heteroclitus*	3.59	Hunting: meiofauna	Copepods	Polychaetes	(Pauly 1989)
Winter Flounder	*Pseudopleuronectes americanus*	3.62	Hunting: meiofauna	Polychaetes	Copepods	(Bowman 2000)
Grubby	*Myoxocephalus aeneus*	3.70	Hunting: macrofauna	Shrimp	Amphipods	(Laroche 1982)
Atlantic Needlefish	*Strongylura marina*	3.78	Hunting: macrofauna	Small fishes	Copepods	(Bowman 2000)
Scup	*Stenotomus chrysops*	3.82	Hunting: macrofauna	Fish	Copepods	(Bowman 2000)
Oyster Toadfish	*Opsanus tau*	3.84	Hunting: macrofauna	Fish	Shrimp	(Adams 1976)
Black Seabass	*Centropristis striata*	3.89	Hunting: macrofauna	Decapods	Fish	(Bowman 2000)

Summer Flounder	*Paralichthys dentatus*	4.49	Hunting: macrofauna	Fish	Squid	(Bowman 2000)
Inshore Lizardfish	*Synodus foetens*	4.50	Hunting: macrofauna	Fish		(Bowman 2000)

Table S2: The statistical output from the correlations between trophic composition matrix with the functional trait community ordination. nMDS1 and nMDS2 show the vector directions and bolded P values indicate significant correlations.

**Species**	**nMDS1**	**nMDS2**	**R^2**	**P value**
Detritovore	-0.0867	-0.9962	0.06	0.302
Planktivore	-0.6223	0.7826	0.25	**0.027**
Hunting: meiofauna	-0.1627	-0.967	0.40	**0.003**
Hunting: macrofauna	0.4131	0.9107	0.03	0.604

Figure S2: Biplot correlating the trophic group community matrix with the functional trait community ordination. The functional trait scores are plotted on the ordination with overlaying correlating vectors of feeding modes. The colored arrow indicates feeding mode vector correlations with the functional trait ordinations which had p-value of $\geq$0.05 and the grey arrows are feeding modes, which had a p-value of $\leq$ 0.05.

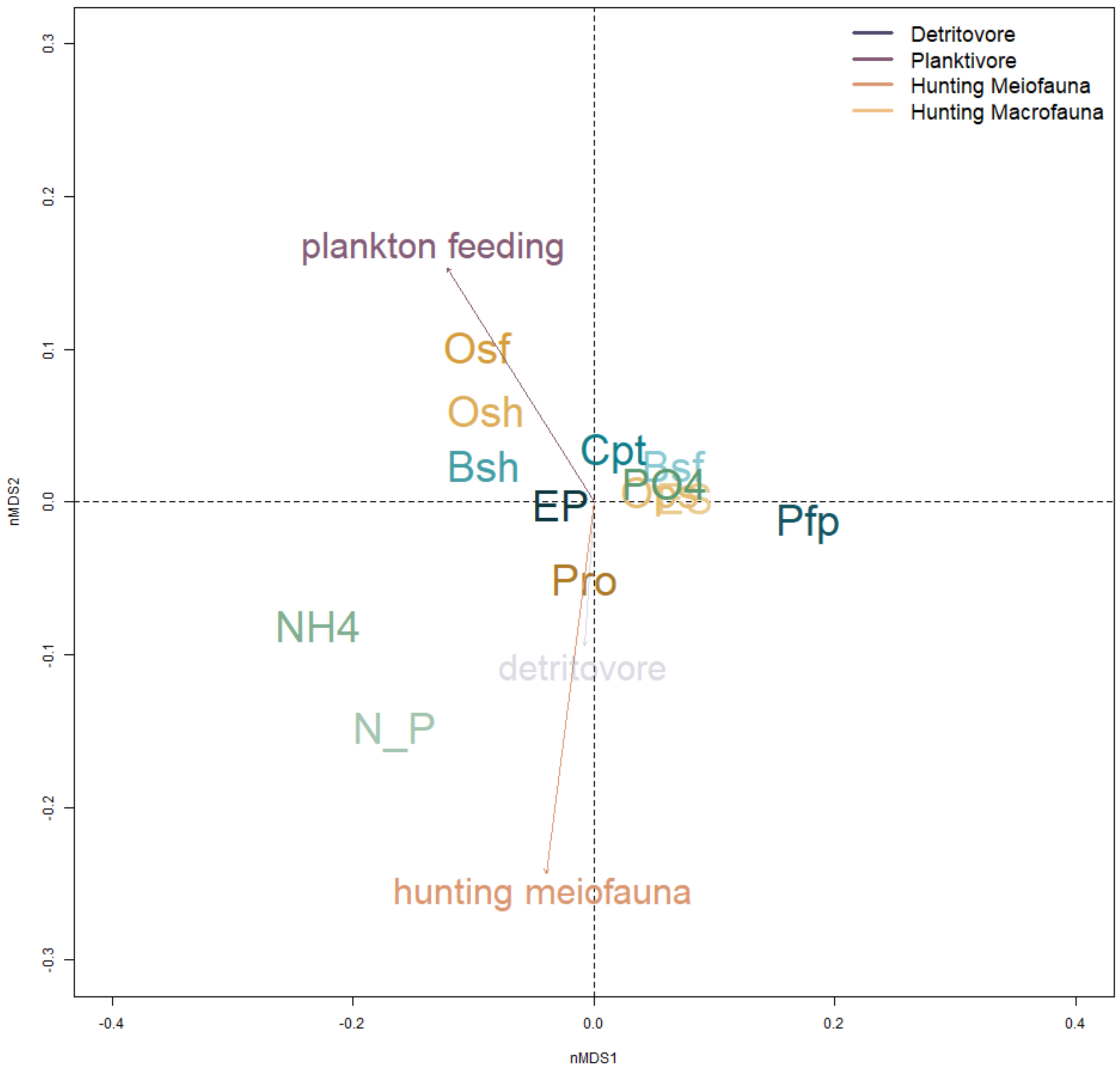

Figure S3. Species abundances and sum of abundances through time for the two most stable pond communities grouped by their functional trophic feeding group. This figure shows that as species within a functional group fluctuate through time, the total sum of abundance remains relatively constant.

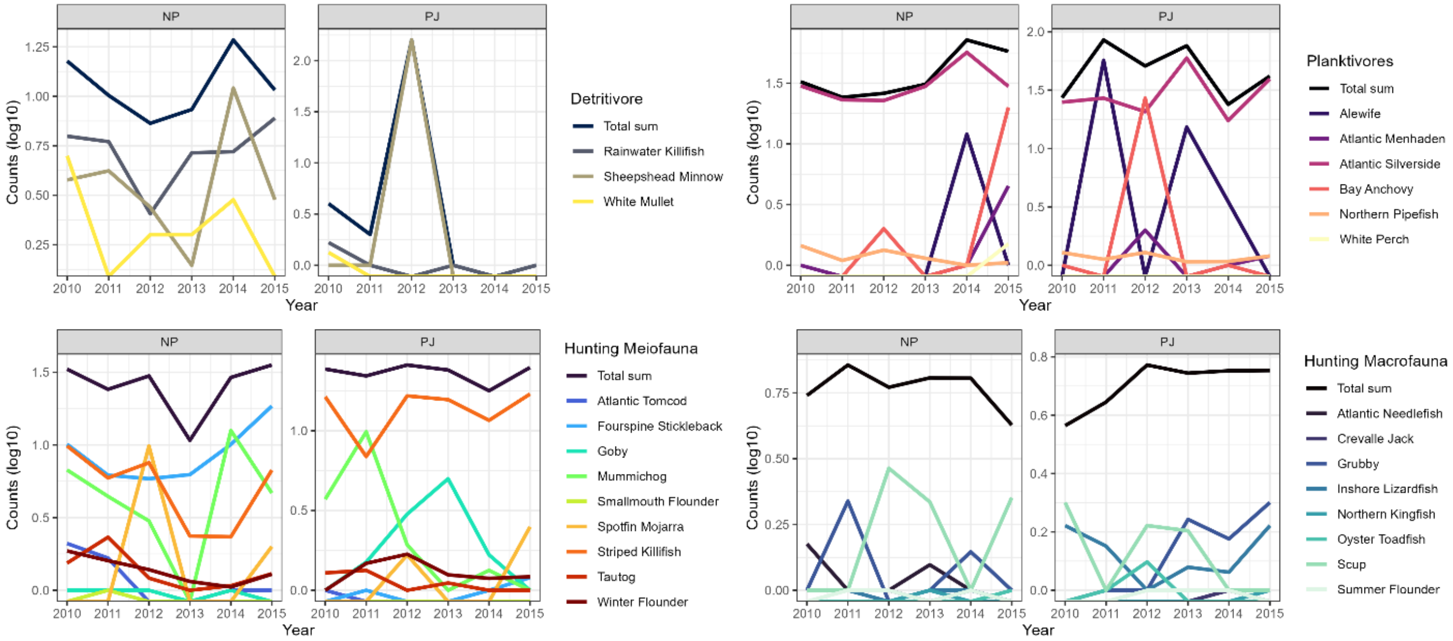

References

Adams, S. M. 1976. Feeding ecology of eelgrass fish communities. Transactions of the American Fisheries Society 105:514–519.

Allen, D. M., W. S. Johnson, and V. Ogburn-Matthews. 1995. Trophic relationships and seasonal utilization of salt-marsh creeks by zooplanktivorous fishes. Environmental biology of fishes 42:37–50.

Austin, H. M., and S. E. Austin. 1971. The feeding habits of some juvenile marine fishes from the mangroves in western Puerto Rico. University of Puerto Rico.

Bowman, R. E. 2000. Food of northwest Atlantic fishes and two common species of squid.

Froese, R. and D. Pauly. Editors. 2023.FishBase. World Wide Web electronic publication. www.fishbase.org, (02/2023).

Gómez-Canchong, P., Manjarrés, L., Duarte, L. O., & Altamar, J. (2004). Atlas pesquero del área norte del Mar Caribe de Colombia. *Universidad del Magdalena, Santa Marta*, *230*.

Grabe, S. A. 1978. Food and feeding habits of juvenile Atlantic tomcod, Microgadus tomcod, from Haverstraw Bay, Hudson River. Fish. Bull 76:89–94.

Harrington, R. W., and E. S. Harrington. 1961. Food Selection among Fishes Invading a High Subtropical Salt Marsh: From Onset of Flooding through the Progress of a Mosquito Brood. Ecology 42:646–666.

José, L.-B. M., and B. J. Olinto. 2003. Alimentação natural de Etropus crossotus Jordan & Gilbert (Teleostei, Pleuronectiformes, Paralichthyidae), na Armação do Itapocoroy, Penha, Santa Catarina, Brasil.

Laroche, J. L. 1982. Trophic patterns among larvae of five species of sculpins (Family: Cottidae) in a Maine estuary [Myoxocephalus aenaeus, Myoxocephalus octodecemspinosus, Myoxocephalus scorpius, Triglops murrayi, Hemitripterus americanus, Damariscotta River]. Fishery bulletin United States, National Marine Fisheries Service.

Leim, A. H. 1966. Fishes of the Atlantic coast of Canada. Bull. Fish. Res. Bd. Can. 155:1–485.

Lewis, V. P., and D. S. Peters. 1994. Diet of Juvenile and Adult Atlantic Menhaden in Estuarine and Coastal Habitats. Transactions of the American Fisheries Society 123:803–810.

Murdy, E. O., R. S. Birdsong, and J. A. Musick. 1997. Fishes of Chesapeake Bay. Smithsonian Institution Press.

Pauly, D. 1989. Food consumption by tropical and temperate fish populations: some generalizations. Journal of Fish Biology 35:11–20.

Prout, M. W., E. L. Mills, and J. L. Forney. 1990. Diet, Growth, and Potential Competitive Interactions between Age-0 White Perch and Yellow Perch in Oneida Lake, New York. Transactions of the American Fisheries Society 119:966–975.
