# Supplementary material for: Functional trait analysis reveals the hidden stability of multitrophic communities
Source: Ecology. 2025 Feb 23;106(2):e70001. doi: 10.1002/ecy.70001 (PMC11848122; doi:10.1002/ecy.70001)

Yeager, M.E., Hughes, A.R. Functional trait analysis reveals the hidden stability of multitrophic communities. Ecology

Appendix S7. Dimensional reduction analysis and conceptual examples of spatiotemporal turnover and the asymmetrical mapping traits within species.

Figure S1. Mean relative stability dissimilarity calculations for the dimensional reduction test. Plots a-d are the mean relative dissimilarity when mock communities were constructed and resampled with 13 species and 13 functional traits. Plots e-h are the mean relative dissimilarity when mock communities were constructed and resampled with 8 species and 8 functional traits. The first two columns (a, b, e, f) are the relative year to centroid dissimilarity and the last two columns (c, d, g, h) are the relative year to year dissimilarity. Each simulation type was resampled 10,000 times.

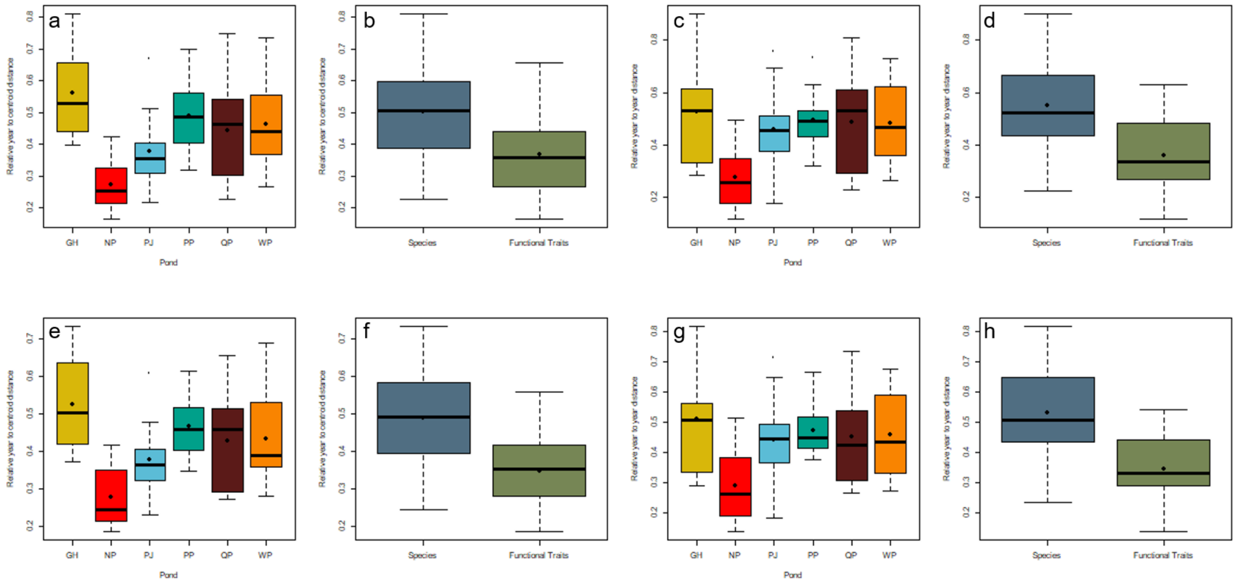

Figure S2. (a) A conceptual figure of the temporal and spatial environmental filters which act on species (N = 12) and functional (N = 4) richness. Examining three hypothetical years across two ponds, we can see that although species vary from year to year within pond, functional trait composition remains, with differences in both species and traits across pond communities. (b) The asymmetrical mapping of species and functional traits. By only capturing a subset of the functional traits (colored in, blue shapes: dark blue for Bluefish, light blue for Black Seabass), we likely miss the functional traits which discriminate species from one another ('missing' absent colored shapes). With the high degree of redundancy based on the traits measured, many species will map onto the same set of functional traits. Image credits as follows: Tracey Zaxby, Integration and Application Network (ian.umces.edu/media-library), for oyster toadfish, bay anchovy, silverside, mummichog, searobin, lizardfish, black seabass and bluefish; Jane Thomas, Integration and Application Network (ian.umces.edu/media-library), for summer flounder, winter flounder; Kim Kraeer and Lucy Van Essen-Fishman, Integration and Application Network (ian.umces.edu/media-library), for alewife; Dieter Tracey, Integration and Application Network (ian.umces.edu/media-library), for white mullet.

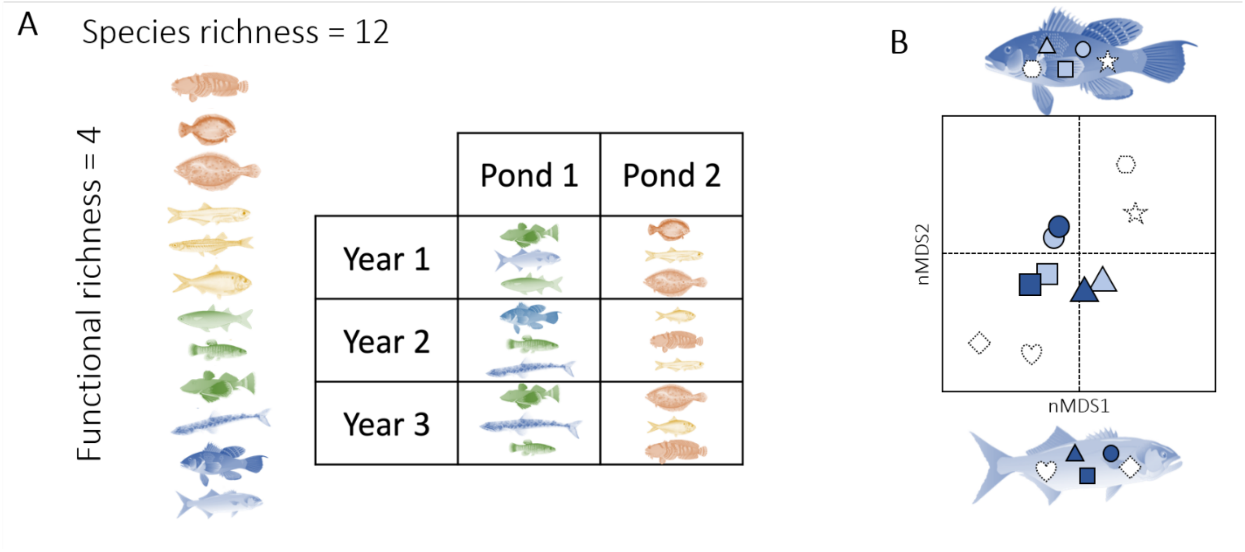

Supplement: Supplementary file 7 — Appendix S7. [file ECY-106-e70001-s005.pdf]
